# Interference between variants of peach latent mosaic viroid reveals novel features of its fitness landscape: implications for detection

**DOI:** 10.1038/srep42825

**Published:** 2017-02-17

**Authors:** Pedro Serra, Edson Bertolini, M. Carmen Martínez, Mariano Cambra, Ricardo Flores

**Affiliations:** 1 Instituto de Biología Molecular y Celular de Plantas, Consejo Superior de Investigaciones Científicas-Universidad Politécnica de Valencia, Spain; 2 Instituto Valenciano de Investigaciones Agrarias, Moncada, Valencia, Spain; 3 Departamento de Fitossanidade, Faculdade de Agronomia, Universidade Federal do Rio Grande do Sul, Porto Alegre, Brazil

## Abstract

Natural populations of peach latent mosaic viroid (PLMVd) are complex mixtures of variants. During routine testing, TaqMan rtRT-PCR and RNA gel-blot hybridization produced discordant results with some PLMVd isolates. Analysis of the corresponding populations showed that they were exclusively composed of variants (of class II) with a structural domain different from that of the reference and many other variants (of class I) targeted by the TaqMan rtRT-PCR probe. Bioassays in peach revealed that a representative PLMVd variant of class II replicated without symptoms, generated a progeny with low nucleotide diversity, and, intriguingly, outcompeted a representative symptomatic variant of class I when co-inoculated in equimolecular amounts. A number of informative positions associated with the higher fitness of variants of class II have been identified, and novel sets of primers and probes for universal or specific TaqMan rtRT-PCR detection of PLMVd variants have been designed and tested.

Viroids, subviral replicons consisting only of a small non-protein-coding RNA, may either incite disease or infect their host plants latently^1^,^2^,^3^,^4^,^5^,^6^,^7^. This is even the case between sequence variants of certain viroids, as illustrated by peach latent mosaic viroid (PLMVd), genus *Pelamoviroid,* family *Avsunviroidae*^8^,^9^. Most PLMVd variants replicate without inciting conspicuous leaf symptoms —the term latent in the name of the viroid refers to this feature— while a few cause mosaics/blotches of different severity and still others an extreme albinism (peach calico)^10^,^11^,^12^,^13^,^14^,^15^,^16^,^17^. Because these phenotypic alterations may fluctuate over time, with a latent strain evolving into symptomatic or, more frequently, the other way around^10^,^11^,^12^, proper diagnosis of field plants and propagation material is crucial to control or mitigate the disease effects.

Before a viroid was identified as the causal agent of peach latent mosaic, the disease was diagnosed by a cross-protection assay in greenhouse-grown GF305 (a peach seedling indicator for most viruses and viroids infecting *Prunus* spp.)^18^. Following identification of PLMVd as a physical entity by a double polyacrylamide gel electrophoresis (PAGE) approach specific for small circular RNAs^19^, this technique was applied for detecting the viroid and fulfilling Koch’s postulates^20^. Following PLMVd cloning and sequencing^8^, more sensitive diagnostic tools including dot-blot hybridization with radioactively- and chemically-labeled full-length riboprobes^21^,^22^,^23^,^24^,^25^, and RT-PCR with specific primers^26^,^27^,^28^,^29^, were developed. Subsequently, several real-time RT-PCR (rtRT-PCR) approaches using different primers and probes were implemented^30^,^31^,^32^. Finally, PLMVd can be also detected with microarrays^33^ and next-generation sequencing^34^,^35^,^36^,^37^.

When routinely testing the presence of PLMVd in commercial peach trees, some that did not react by TaqMan rtRT-PCR produced a clear signal by RNA gel-blot hybridization. This unexpected observation, given the higher sensitivity of the former approach, prompted a search that resulted in the finding of PLMVd isolates composed exclusively of variants with specific sequence changes with respect to those of PLMVd isolates characterized initially. The changes, nevertheless, preserved the global conformation of the viroid RNA as well as key elements of its higher-order structure. Importantly, some of the changes mapped at the RNA segment used to synthesize the TaqMan probe, thus explaining the negative results observed. The new PLMVd isolates displayed relatively low internal genetic heterogeneity, and GF305 peach seedlings infected with one representative variant of these isolates expressed no symptoms. Moreover, in co-inoculation experiments, this variant outcompeted one previously characterized symptomatic variant (both in the resulting progeny and in the associated phenotype), thus denoting the higher biological fitness of the former. Although we have designed a novel set of primers and probes able to detect by TaqMan rtRT-PCR the two classes of PLMVd isolates, our results warn of the risks of diagnosis tests based on just a small sequence fragment of the pathogen genome, and attest to the need for further periodic validation with another alternative approach.

## Results

### TaqMan rtRT-PCR and RNA gel-blot hybridization show discordant results with certain PLMVd isolates

In initial experiments^10^ in which full-length PLMVd-cDNA clones were prepared by RT-PCR with a pair of primers overlapping a *PstI* site delimited by positions 91 to 135 of the PLMVd reference variant —GenBank accession number M83545.1^8^ with two minor corrections^10^— a region of low variability between positions 140 and 270 was found (hereafter numbering refers to the reference variant). This region served for designing primers RF43 and RF44 (Supplementary Table S1), which were used in the characterization of PLMVd isolates and progeny variants^10^,^11^. A subsequent study confirmed the low variability of the region between positions 140 and 270^38^, thus reinforcing its potential use for detection. Moreover, several *in vitro* approaches revealed a kissing-loop interaction in the same region of the PLMVd (+) strand^39^,^40^,^41^. Such tertiary structural element, which is critical for the *in vivo* viability of another viroid of the same genus^42^ and presumably also for PLMVd^40^, should impose additional restrictions to the sequence variability.

**Figure 1:**
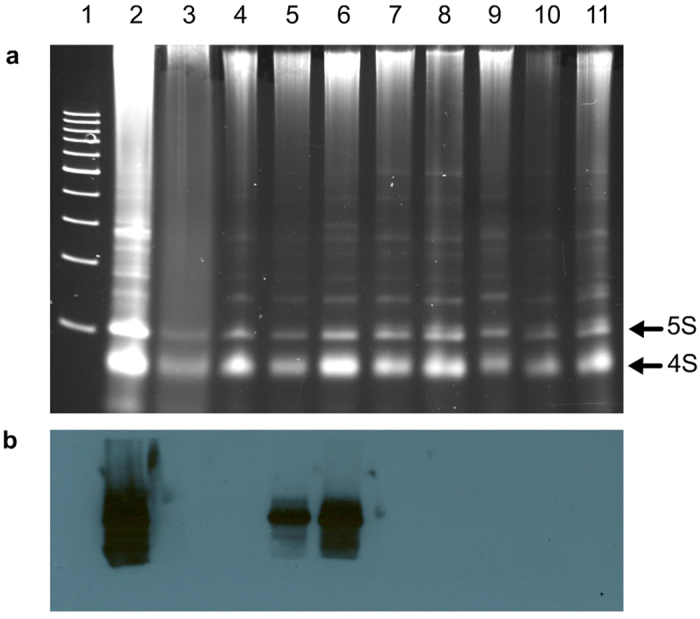
Analysis by molecular hybridization for detecting PLMVd in commercial peach samples. **(a)** Non-denaturing electrophoresis in a 5% polyacrylamide gel stained with ethidium bromide. Arrows on the right indicate the bands generated by 5S and 4S RNAs. **(b)** RNA gel-blot hybridization, with a full-length digoxigenin-labeled riboprobe for detecting PLMVd (+) strands, of the RNAs migrating in gel segment delimited by the 200- and 500-bp DNA size markers. Lane 1, 100-bp DNA multimers size markers. Lanes 2 and 3, RNA preparations from PLMVd-infected and mock-inoculated GF305 peach seedlings, respectively. Lanes 4 to 11, RNA preparations from commercial peach trees. The intense hybridization signals observed in the positive control (lane 2) and in two of the samples (V1 and V2, lanes 5 and 6, respectively) correspond to a size around the 300-bp marker. Even if the RNA amount loaded in the mock-inoculated control (lane 3) was lower than in some samples (e.g. lane 6), it was similar to another one that generated a strong hybridization signal (lane 5).

Accordingly, examination for PLMVd in commercial peach cultivars has been performed at the Instituto Valenciano de Investigaciones Agrarias (IVIA, Spain) by an rtRT-PCR approach based on TaqMan chemistry using two primers, RP1 and FP1, and a fluorescent probe P1 (Supplementary Table S1), derived from the PLMVd region displaying low variability. TaqMan rtRT-PCR is a specific, sensitive and simple approach particularly adaptable to massive analysis^43^. Periodic assessment with a second technique showed that, unexpectedly, two samples (V1 and V2) that did not react by rtRT-PCR produced strong signals by RNA gel-blot hybridization with a full-length riboprobe for detecting PLMVd (+) strands (Fig. 1b, lanes 5 and 6). This discrepancy was neither due to poor nucleic acid extraction, because the non-denaturing polyacrylamide gel stained with ethidium bromide revealed the cellular 5S and 4S RNAs in all samples (Fig. 1a), nor to the presence in the RNA preparations of DNA polymerase inhibitors blocking rtRT-PCR, because the PLMVd-infected GF305 peach seedling serving as control reacted positively (data not shown). Moreover, considering that conventional RT-PCR with the same two primers used in rtRT-PCR amplified a PLMVd fragment of the expected length (data not shown), we concluded that the probe was most likely responsible for the negative results obtained by rtRT-PCR with samples V1 and V2. As a corollary, given the high sensitivity of rtRT-PCR, these two samples should contain only variants not recognized by the probe.

### The novel PLMVd isolates are exclusively composed of variants with a structural domain different from that of the reference and many other variant

**Figure 2:**
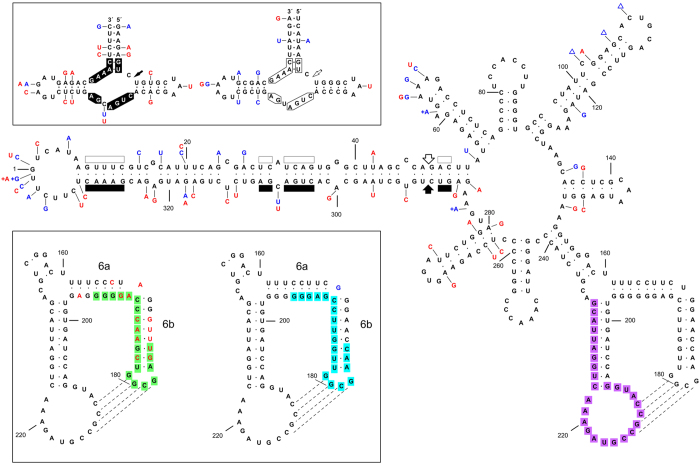
Primary and proposed secondary structure for the PLMVd plus strand of the reference variant GenBank M83545. 1^8^ with two minor corrections^10^. Changes in the representative symptomatic variant of class I (gds6)^10^ and in the representative variant of class II (v1.1) (this work), are denoted with blue and red characters, respectively. Symbols (+) and (Δ) refer to insertions and deletions, respectively, and broken lines to the kisssing-loop interaction^39^. *Upper inset,* hammerhead structures of the PLMVd plus and minus strands with the self-cleavage sites marked with arrows. Substitutions in variants gds6 and v1.1 do not disrupt the helices flanking the central core of 13 nucleotide residues (boxed) conserved in most natural hammerhead structures of viroid and viroid-like satellite RNAs. The same numbering is used for both polarities. *Lower inset,* domain 6a/6b harboring characteristic changes between variants of both classes. Note that helix 6a of variant v1.1 is one base-pair shorter. Fragments covered by the TaqMan rtRT-PCR probes recognizing variants of class I, class II, and of both classes, are indicated with green, blue and purple backgrounds, respectively. Sequence changes in variants gds6 and v1.1 may result in minor rearrangements of the secondary structure not represented here (see Supplementary Fig. S1).

To find an explanation for the discordant results obtained with the two techniques for samples V1 and V2, we performed RT-PCR amplifications of the corresponding RNA preparations with primers RF43 and RF44 followed by cloning and sequencing of the full-length products. Analysis of 10 clones of each sample showed that they were just composed of variants with multiple changes in the region delimited by positions 161–197, forming the so-called stems P6a and P6b^39^, the latter capped by one of the loops involved in the kissing-loop interaction (Fig. 2). This observation accounted for the negative results obtained by rtRT-PCR, considering that probe P1 (positions 175–193) mapped at a segment of this region with several substitutions with respect to the reference variant (Fig. 2).

To better characterize the population structure of samples V1 and V2, particularly in the region covered by primers RF43 and RF44, we performed a second RT-PCR amplification with another pair of adjacent primers of opposite polarity (RF1251 and RF1252) (Supplementary Table S1), designed on a region of low variability observed in variants of the V1 and V2 populations obtained with the first primer pair. Cloning and sequencing of the full-length products from the second amplification, 10 clones from each sample, corroborated and expanded the previous results by showing additional changes in stems P6a and P6b with respect to the reference variant. Interestingly, the same changes were observed in all variants: co-variations or replacement of a canonical by a wobble pair (or *vice versa)* that did not disrupt the double-stranded structure of stems P6a and P6b and, therefore, the kissing-loop interaction (Fig. 2). Direct sequencing of the amplification product confirmed without ambiguities the same sequence for the region P6a/P6b (data not shown), thus reinforcing its lack of variability. On the basis of the different P6a/P6b stems, hereafter we will refer to V1 and V2 variants as class II variants to discriminate them from those more frequent of class I, like the reference and many other variants including gds6 (see below). Although the sequence changes of class II variants in one strand of stem P6b reduced the stability of the hybrid formed by primer RF43 and the RNA template, it did not preclude RT-PCR amplification because no changes were observed in those positions complementary to the nine 3′-terminal nucleotides of this primer.

The changes observed in V1 and V2 variants neither affected the nucleotides conserved in most natural hammerhead structures of viroid and viroid-like satellite RNAs^44^,^45^,^46^ nor their thermodynamic stability, because they mapped at loops or, when found in the stems, they were co-variations replacing a canonic base-pair by another or by a wobble pair (Fig. 2 and Supplementary Fig. S1). Hammerhead structures are ribozymes that play a key role mediating the self-cleavage of the oligomeric RNA strands generated in the rolling-circle replication of members of the family *Avsunviroidae* and of viroid-like satellite RNAs^47^,^48^. Furthermore, the changes in V1 and V2 variants did not have major effects on the secondary structure of minimal free energy predicted for the PLMVd (+) strand, preserving the long hammerhead arm^10^, although some local elements of secondary structure differed from those of the reference variant (Supplementary Fig. S1). A similar overall conformation is supported by selective 2′-hydroxyl acylation analyzed by primer extension (SHAPE) *in vitro*^49^. Regarding the global secondary structure of the PLMVd (−) strand proposed here (Supplementary Fig. S2), the co-variations detected between the reference and gds6 variants (both of class I) and the representative variant of class II (v1.1) (see below) are more consistent with a conformation proposed recently^49^ than with another presented previously^40^,^41^, in both instances obtained by SHAPE *in vitro.* Therefore, one single secondary structure of the PLMVd (−) strand (Supplementary Fig. S2) accounts for the variability observed in variants of the two classes.

**Figure 3:**
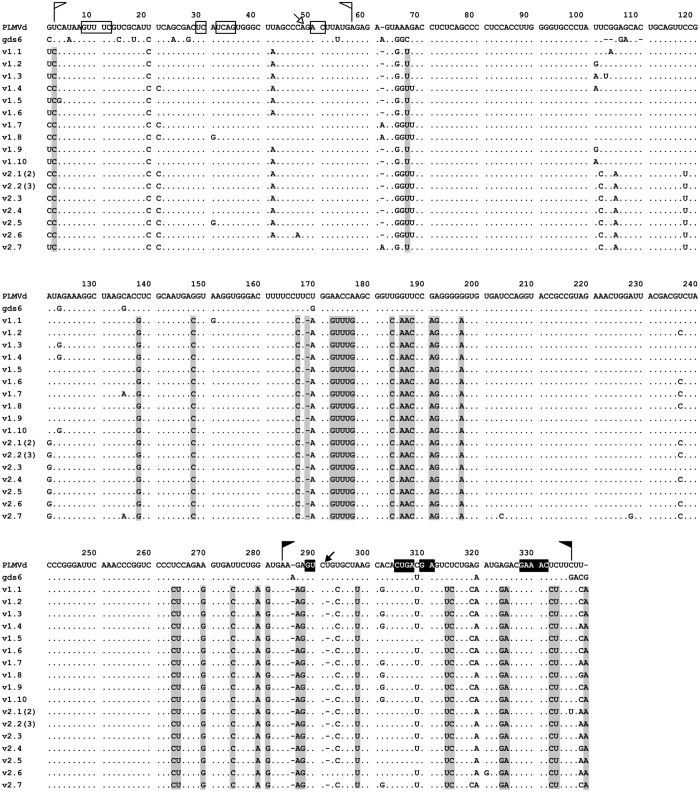
Alignment of the 17 different variants obtained from two PLMVd isolates of class II (V1 and V2) by RT-PCR using the pair of adjacent primers of opposite polarity RF1251 and RF1252 (Supplementary Table S1). The reference and gds6 variants (of class I), included for comparative purposes, appear at the top. Dashes denote gaps and dots nucleotide identity with respect to the reference sequence. Regions forming the plus and minus hammerhead structures are flanked by flags, the nucleotide residues conserved in most natural hammerhead structures of viroid and viroid-like satellite RNAs are within boxes, and the self-cleavage sites are shown by arrows; black and white backgrounds refer to plus and minus polarities, respectively. Informative positions of the 17 variants of class II, with respect to the reference and gds6 variants of class I, are with grey background. When the same variant was recovered more than once it is indicated between parentheses. Other details as in the legend to Fig. 2.

To better define and map the variability of samples V1 and V2, we next performed an alignment of the 17 different variants of class II obtained with primers RF1251 and RF1252 (variants 2.1 and 2.2 were recovered two and three times, respectively), and extracted their characteristic informative positions when compared to the reference and gds6 variants of class I. These informative positions mostly include single to up to five consecutive nucleotide substitutions, and they are distributed throughout the whole molecule (Fig. 3). The estimated nucleotide diversity was 0.0273+/−0.0002.

Finally, to detect specifically variants of class II by TaqMan rtRT-PCR with the same pair of primers used before, FP1 and RP1, we designed a novel probe P2 covering the same positions (175–193) as the previous one, but identical to the sequence of class II variants (Supplementary Table S1). Such specific detection was confirmed in experiments with singly-inoculated and with co-inoculated variants of both classes (see below).

### A representative PLMVd variant of class II replicates without symptoms, generates a progeny with low nucleotide diversity, and outcompetes a representative symptomatic variant of class I when co-inoculated

To examine the biological properties of class II variants, we focused on the 17 different ones obtained from samples V1 and V2 with primers RF1251 and RF1252, (Supplementary Table S1) (GenBank accession numbers KX430152 to KX430168), and selected as representative of class II one of the variants more similar to the consensus sequence of the series (Fig. 3). Importantly, this representative variant (v1.1) (GenBank accession number KX430152) does not have the deletion of the U located 3′ to the self-cleavage site of the plus hammerhead structure. Such deletion, observed in some PLMVd variants previously and in the present work, strongly reduces the self-cleavage activity and the infectivity^10^,^11^, and it is most likely an artifact introduced by the reverse transcriptase^50^.

First, in a preliminary bioassay, dimeric head-to-tail *in vitro* transcripts of variant v1.1 were mechanically inoculated into 10 GF305 peach seedlings, with four control seedlings being mock-inoculated with buffer. RNA gel-blot hybridization of preparations from leaves collected four months later revealed that the 10 plants inoculated with variant v1.1 were infected (data not shown), thus attesting to its high infectivity. However, none expressed any differential phenotype with respect to that of the mock-inoculated controls, suggesting that variant v1.1 was latent. Moreover, RT-PCR with primers RF1251 and RF1252 (Supplementary Table S1), cloning and sequencing of 10 clones (five from one plant and five from a second one) showed that all were of class II (data not shown).

To confirm that variant v1.1 was indeed latent and to examine another biological features thereof, we performed a second bioassay in which three blocks of 10 GF305 seedlings each were singly-inoculated with equal amounts of dimeric transcripts of variants v1.1 or gds6 (the latter a representative variant of class I that induces a severe mosaic)^10^,^11^, and co-inoculated with equal amounts of dimeric transcripts of the two variants; six mock-inoculated GF305 seedlings served as controls. When three months post-inoculation gds6-inoculated plants were examined by RNA gel-blot hybridization and TaqMan rtRT-PCR with primers FP1 and RP1 and probe P1 (Supplementary Table S1), only 2/10 plants tested positive (Table 1). In contrast, 8/10 plants inoculated with variant v1.1 were infected according to RNA gel-blot hybridization and rtRT-PCR with the same primers and the second probe (P2); the discrepancy between both approaches observed for the two remaining plants (Table 1, number 4 and 5) most likely resulted from the higher sensitivity of rtRT-PCR. As anticipated, none of the seedlings singly-inoculated with variants gds6 and v1.1 reacted with probes P2 and P1, respectively (the high threshold cycle, Ct, of number 8 with P1 suggests that a minor contamination occurred during analysis), showing that such probes were indeed class-specific (Table 1). Altogether these data supported that variant v1.1 was more infectious than gds6. On the other hand, in the co-inoculated block, 5/10 plants tested positive by RNA gel-blot hybridization; interestingly, they and at least four more plants (Table 1, number 15, 18, 19 and 20) reacted clearly by rtRT-PCR, but only with probe P2 for class II variants (while no signal was generated with probe P1 for class I variants) (Table 1). These results confirmed the higher infectivity of variant v1.1 and, unexpectedly, they also revealed that it was able to fully outcompete variant gds6.

When the same 30 plants were assessed five months post-inoculation, every one generated strong signals by RNA gel-blot hybridization. In agreement with such results: i) all plants of the two blocks of 10 seedlings singly-inoculated with variants gds6 and v1.1 tested positive by rtRT-PCR, but only with the probes specific for class I and II variants, respectively, and ii) all plants of the block of 10 seedlings co-inoculated with variants gds6 and v1.1 tested positive by rtRT-PCR, but only with the probe specific for class II variants (Table 1). These data further corroborated the ability of variant v1.1 to exclude variant gds6. The exclusion did not result from their different accumulation levels, as revealed by the Ct values at five months post-inoculation (once infection was established) (Table 1).

Because after eight months in the greenhouse the plants —including those inoculated with the mosaic-inducing variant gds6— did not express symptoms, they were pruned, chilled at 4 °C for one month in darkness and then transferred back to the greenhouse to stimulate growth of new flushes. While all seedlings singly-inoculated with variant gds6 displayed the expected symptoms in the emerging leaves, phenotypic alterations were neither observed in any of the mock-inoculated seedlings nor in those inoculated with variant v1.1, thus showing that the latter variant was indeed latent as previously suspected. Moreover, none of the seedlings co-inoculated with variants gds6 and v1.1 expressed the typical symptoms associated with gds6, consistent with the absence of gds6-like variants in the resulting progeny. To better document this point, following RT-PCR with another pair of adjacent primers of opposite polarity RF1332 and RF1333 (Supplementary Table S1) and cloning, we sequenced 10 clones from one seedling singly-inoculated with variant gds6, 10 clones from one seedling singly-inoculated with variant v1.1, and 12 clones from four seedlings (three clones from each) co-inoculated with both variants. Subsequent analysis revealed that, as expected, all clones from the seedling singly-inoculated with variant gds6 were of class I and different from each other (nucleotide diversity 0.02716+/−0.00007), while all clones from the seedling singly-inoculated with variant v1.1 were of class II and 8/10 different from each other (nucleotide diversity 0.0173+/−0.0001) (GenBank accession numbers KX430169 to KX430186). On the other hand, all clones from the seedlings co-inoculated with both variants were of class II and 10/12 different from each other (nucleotide diversity 0.0228+/−0.0002). These results showed a lower genetic diversity in the progeny of variant v1.1, and they further confirmed that variants of each class did not evolve one into the other, and that variant v1.1 had a higher fitness than variant gds6 leading to its exclusion when co-inoculated.

### Design of a new set of primers and probe for detecting PLMVd variants of class I and II by TaqMan rtRT-PCR

Finally, with the information gained from the previous experiments, we went back to circumvent the original discrepancy between results from RNA gel-blot hybridization and TaqMan rtRT-PCR by implementing changes to adapt the latter for detecting variants of both classes. As a first step, we performed an alignment of the full-length PLMVd variants reported here and in the literature excluding: i) those with changes affecting the conserved motifs of the hammerhead structures that would turn them non-functional, and ii) the progeny (3,939 different variants) of a single variant of class II examined by next-generation sequencing^36^ using an approach prone to generate sequencing artifacts when compared to conventional Sanger sequencing. Based on this curated and non-redundant alignment (394 sequences) (Supplementary Data file S1), we chose three regions with low variability for designing primers RP2 and FP2 and probe P3 (Supplementary Table S1).

Then, we re-examined with the novel primers and probe the three blocks of 10 GF305 seedlings that were singly-inoculated with equal amounts of dimeric transcripts of variants gds6 and v1.1, and co-inoculated with equal amounts of dimeric transcripts of the two variants. The samples from each plant were those taken 5 months after inoculation. All plants tested positive with a low Ct (Table 1), thus showing the ability of primers RP2 and FP2, in combination with probe P3, to sensitively detect PLMVd variants of class I and II.

## Discussion

The failure of TaqMan rtRT-PCR to detect PLMVd in samples producing strong signals by RNA gel-blot hybridization with a full-length riboprobe was unexpected because, even if PLMVd isolates examined previously in detail^10^,^12^,^13^,^15^,^16^,^17^,^38^ display high sequence variability consistent with a quasispecies model^11^,^36^,^51^,^52^, the extreme sensitivity of TaqMan rtRT-PCR would anticipate detection of low-abundant variants. Cloning and sequencing of two isolates, V1 and V2, with such atypical behavior showed the exclusive existence of variants (here termed of class II) with a P6a/P6b domain different from that initially found in the reference and in many other variants (here termed of class I). PLMVd variants of class II have been reported previously^35^,^37^,^53^,^54^, but their properties have not been thoroughly assessed. Our observation that isolates V1 and V2 are exclusively composed of variants of class II explains the unexpected results —given that the initial TaqMan probe (P1) targets a fragment of domain P6a/P6b of class I variants— and, most importantly, indicates a strong interference/exclusion between variants of both classes. This assumption was confirmed in co-inoculations with representative variants of class I (gds6) and class II (v1.1): the latter, possibly due to its higher infectivity (detected soon after inoculation), outcompeted the former. Such results are in line with those of the original cross-protection tests, wherein pre-inoculated latent strains protected GF305 peach seedlings against challenge inoculations with a severe strain^18^. However, some important distinctions should be made. Specifically, in the bioassay reported here: i) variants, not strains composed of many variants, were used, ii) variants were co-inoculated, and not inoculated one after the other, and iii) the resulting progeny, and not only the presence or absence of symptoms, was analyzed. Furthermore, in a different experimental context, when the sectors of the same symptomatic leaf expressing peach calico (PC) were dissected from the adjacent green tissues before extraction, the respective PLMVd progeny was enriched in the characteristic PC- and non-PC-inducing variants, respectively^13^,^15^, thus indicating strong competition between variants during host colonization. Hence, interference/exclusion between PLMVd variants seems a general phenomenon.

Analysis of the population structure of other PLMVd isolates reported previously also supports interference/exclusion between class variants I and II in natural infections: either they do not co-exist^10^,^12^,^13^,^16^,^54^ (and this work) or when they do, variants of class I were detected at low frequency in populations of class II variants and, less frequently, the reverse situation^35^,^37^,^53^. This low frequency might be artifactual or, at least in some cases, result from erroneously annotated sequences. Moreover, under field conditions, trees may become re-infected as a result of diverse agronomic practices, thus complicating interpretation of the results.

Interference between viroids, or strains of the same viroid, has been noticed before^55^ (for a review see ref. 2). Pertinent to the present context is the interference reported between variants of chrysanthemum chlorotic mottle viroid (CChMVd), grouped with PLMVd into the genus *Pelamoviroid*^56^. Co-inoculations with equal amounts of two CChMVd representative variants —one symptomatic and the other latent, differing in a tetraloop with which pathogenicity is strictly associated— resulted in a progeny in which variants of both classes accumulated in a ratio 3:1 in favor of that associated with symptoms^57^. Thus, in contrast with the PLMVd case, complete exclusion was not observed in CChMVd, at least along the period covered in the experiment. The mechanism mediating this partial/total exclusion is not known, but there is some evidence supporting the involvement of RNA silencing^2^,^58^.

It is worth noting that in the progenies of variants gds6 and v1.1 (and in the populations of isolates V1 and V2) here reported, we have not found single co-variations or replacements of a canonical by a wobble pair (or *vice versa)* in the P6a/P6b domain. Moreover, a similar situation is predominantly observed in other full-length PLMVd variants reported previously (Supplementary Data file S1): they have the P6a/P6b domain of either gds6 or v1.1 with some minor exceptions. For instance, three single co-variations and one double co-variation have been independently observed in the P6b stem of different variants of class I (Table 2); however, such co-variations do not represent intermediate stages in the evolution of the P6b stem of variants of class I into the P6b stem of variants of class II because they do not appear in the latter. Altogether these data suggest that besides the involvement of the secondary structure of domain P6a/P6b in facilitating the kissing-loop interaction (Fig. 2), the sequence of this domain plays also a role in replication, movement or another key function ultimately resulting in the higher biological fitness of class II variants. We cannot rule out, however, that other informative changes discriminating variants gds6 and v1.1 (Fig. 3) may contribute to the higher fitness of the latter, although we deem unlikely that these changes, by themselves, could determine the different fitness. A clearer response to this issue, and to the differential pathogenicity of variants gds6 and v1.1, is very difficult at this stage given the high rate at which PLMVd accumulates variability^11^,^36^. On the other hand, the lack of intermediate P6a/P6b domains between those of gds6 and v1.1, makes it difficult to conceive how variants of one class have evolved from the other; perhaps the intermediate P6a/P6b domains have just a transient existence. Moreover, given the higher fitness displayed by variant v1.1 in the competition experiment with gds6, evolution of class II variants from those of class I appears more feasible than the other way around. It is even possible that variants of both classes may have evolved independently from a common ancestor.

As a final point we would like to stress that our data, in addition to unveiling novel features in the fitness landscape of PLMVd, also alert on the risks intrinsic to detection methods, like TaqMan rtRT-PCR, based on a short fragment of the target RNA. The high specificity of this approach is its main advantage but also its “Achilles’ heel”. Another TaqMan rtRT-PCR approach^30^ may suffer similar constraints because the probe also maps at the PLMVd domain P6a/P6b. However, the TaqMan rtRT-PCR alternatives proposed previously^32^ and here, as well as another rtRT-PCR using a different detection technology (SYBR Green)^31^, should avoid the problem. In any case, our results stress the need of periodic validation of rtRT-PCR approaches with a second independent technique as recommended by OEPP/EPPO (www.eppo.org).

## Materials and Methods

### Plant material and bioassay

Young leaves were collected in Catalonia (Spain) from nursery-grown peach trees *(Prunus persica* L. Batsch) of different commercial varieties, and from greenhouse-grown GF305 peach seedlings inoculated by repeated stem slashing with dimeric head-to-tail *in vitro* transcripts (approximately 1 μg per plant) from specific PLMVd variants (resuspended in 50 mM K_2_HPO_4_) or mock-inoculated with buffer^10^. GF305 seedlings were examined weekly for symptom expression in emerging leaves. When indicated, the seedlings were pruned, chilled at 4 °C in darkness for one month, and moved back to the greenhouse to promote growth of new shoots, the leaves of which were observed for symptom appearance.

### RNA extraction, fractionation, and analysis by PAGE and molecular hybridization

Total nucleic acids were extracted with buffer-saturated phenol and fractions enriched in viroid RNAs were obtained by partitioning with non-ionic cellulose as reported previously^19^. Aliquots were electrophoresed in non-denaturing 5% polyacrylamide gels prepared and run in 1X TAE (40 mM Tris, 20 mM acetate, 1 mM EDTA, pH 7.2 with acetic acid) that were stained with ethidium bromide. RNAs were subsequently electrotransferred to positively-charged nylon membranes (Amersham Hybond-N), hybridized at 70 °C in the presence of 50% formamide with a full-length digoxigenin-labeled RNA probe for detecting PLMVd (+) strands^21^, and revealed as recommended by the supplier (Roche).

### RT-PCR amplification, cloning, and sequencing

PLMVd-enriched preparations, or the monomeric forms eluted from polyacrylamide gels, were incubated with Moloney murine leukemia virus reverse transcriptase (RT Superscript II, Invitrogen), and the cDNA of an aliquot (1/20) of the RT reaction mixture was PCR-amplified with *Pfu* DNA polymerase (Agilent) essentially as described previously^8^,^10^ but using the primer pairs indicated in Results and Supplementary Table S1. The ensuing products were separated by non-denaturing PAGE in 5% gels, and the PLMVd-cDNAs of the expected full-length were eluted and ligated in the *EcoR*V restriction site of plasmid pBSII KS+(Stratagene). The resulting recombinant plasmids were cloned in competent *Escherichia coli* cells (DH5α), and their inserts sequenced by capillary electrophoresis using an ABI 3130XL apparatus (Life Technologies) and a Big Dye Terminator v3.1 cycle sequencing kit (Applied Biosystems). Alignments were performed with the ClustalW program within the MEGA integrated tool version 6.06^59^, and the nucleotide diversity (mean and variance) of isolates and progeny was estimated as indicated previously^11^,^60^ using also MEGA. For infectivity bioassays, the monomeric PLMVd-cDNA inserts of some specific recombinant plasmids were PCR-amplified with the same (previously phosphorylated) primers used in their generation. The resulting fragments of the expected full-length were gel-eluted and treated with DNA ligase (Fermentas) to produce head-to-tail dimers that were subsequently cloned in the same vector (pBSII KS+) and sequenced to confirm orientation and lack of artifactual mutations. The PLMVd nucleotide sequences obtained in this study have been deposited in GenBank (accession numbers KX430152 to KX430176).

### Detection by TaqMan rtRT-PCR

Sample extraction and subsequent examination were essentially as reported previously^43^,^61^. In brief, leaf tissue was ground with a Homex-6 homogenizer (Bioreba) and extraction buffer (PBS pH 7.2, containing 0.2% sodium diethyldithiocarbamate and 2% polyvinil-pyrrolidone PVP-10) in a relation 1:20 (w/v), and an aliquot corresponding to 10 mg of fresh tissue was processed with a PowerPlant^®^ RNA isolation kit (MO BIO) resulting in 50 μl of a partially purified RNA preparation. The reaction mixture (12 μl final volume) contained 1X AgPath-ID One-step RT-PCR master mix (Ambion), 1X RT-PCR enzyme mix (Ambion), 0.5 μM of each primer and 200 nM of the TaqMan probe, and 3 μl of the RNA preparation. The cycling profile consisted of one step of 45 °C for 10 min and 95 °C for 10 min, followed by 40 cycles of amplification (95 °C for 15 s and 60 °C for 1 min). TaqMan rtRT-PCR was performed with a StepOne Plus apparatus (Applied Biosystem) and the corresponding software. TaqMan probes were labeled with the fluorescent dye 6-carboxyfluoroscein (FAM) in the 5′-end, and the quencher N,N,N′,N′-tetramethyl-6-carboxyrhodamine (TAMRA) in the 3′-end.

## Additional Information

**How to cite this article**: Serra, P. *et al*. Interference between variants of peach latent mosaic viroid reveals novel features of its fitness landscape: implications for detection. *Sci. Rep.* **7**, 42825; doi: 10.1038/srep42825 (2017).

**Publisher's note:** Springer Nature remains neutral with regard to jurisdictional claims in published maps and institutional affiliations.

## Supplementary Material

Supplementary Tables and Figures

## Figures and Tables

**Table 1:** Detection of PLMVd in singly-inoculated and co-inoculated GF305 peach seedlings with two independent techniques.

Plant code	Variant inoculated	3 months p.i.			5 months p.i.			
		rtRT-PCR		Gel-blot hybridization	rtRT-PCR			Gel-blot hybridization
		Probe P1	Probe P2		Probe P1	Probe P2	Probe P3	
1	v1.1	−a	12.82^b^	+++^c^	−	12.06	12.99	+++
2	v1.1	−	13.13	+++	−	11.62	12.81	+++
3	v1.1	−	12.71	+++	−	10.62	12.97	+++
4	v1.1	−	14.66	−	−	12.92	14.11	+++
5	v1.1	−	27.52	−	−	12.13	13.79	+++
6	v1.1	−	12.56	+++	−	12.61	13.82	+++
7	v1.1	−	20.68	+++	−	12.04	12.97	+++
8	v1.1	31.21	27.53	+	−	12.03	12.83	+++
9	v1.1	−	12.67	+++	−	10.99	12.96	+++
10	v1.1	−	13.92	+	−	12.94	13.92	+++
11	v1.1 + gds6	−	25.14	−	−	12.16	12.96	+++
12	v1.1 + gds6	−	13.24	+++	−	11.80	13.53	+++
13	v1.1 + gds6	−	12.58	+++	34.15	11.30	14.09	+++
14	v1.1 + gds6	−	12.96	+++	−	12.10	13.59	+++
15	v1.1 + gds6	−	20.31	−	−	12.78	13.98	+++
16	v1.1 + gds6	−	12.79	+++	−	12.13	13.06	+++
17	v1.1 + gds6	−	12.72	+++	23.17	12.57	13.35	+++
18	v1.1 + gds6	−	18.59	−	31.52	12.68	13.90	+++
19	v1.1 + gds6	−	15.65	−	−	11.99	13.63	+++
20	v1.1 + gds6	−	14.77	−	24.22	13.13	13.46	+++
21	gds6	−	−	−	13.56	−	13.83	+++
22	gds6	−	−	−	15.55	−	16.67	+++
23	gds6	−	−	−	13.31	−	14.62	+++
24	gds6	15.18	−	+++	13.79	−	15.37	+++
25	gds6	29.53	−	−	16.31	−	17.67	+++
26	gds6	14.57	−	++	13.71	−	14.93	+++
27	gds6	31.87	−	−	16.21	−	16.91	+++
28	gds6	33.58	−	−	13.82	−	14.89	+++
29	gds6	32.79	−	−	13.58	−	14.44	+++
30	gds6	32.89	−	−	18.44	−	18.85	+++
31	Mock	−	−	−	−	−	34.67^d^	−
32	Mock	−	−	−	−	−	34.19^d^	−
33	Mock	−	−	−	−	−	35.72^d^	−

^a^Undetected.

^b^Threshold cycle (Ct).

^c^Signal intensity from weak (+) to strong (+++).

^d^The high Ct values suggest a minor contamination during analysis because no signal was detected with probe P3 in non-inoculated controls of *Prunus persica. P. dulcis. P. avium* and *P. armeniaca.*

**Table 2:** Single and double co-variations reported in the P6b stem.

Reference variant	Co-variation	Source
U_183_–A_176_	A_183_–U_176_	Hassen *et al*.^53^ Hadidi *et al*.^24^ Gazel *et al*.^54^ Yazarlou *et al*.^16^ Pechalt *et al*.^38^
U_186_–A_173_	G_186_–C_173_	Hassen *et al*.^53^
U_187_–A_172_	G_187_–C_172_	Hassen *et al*.^53^
G_188_–C_171_ U_187_–A_172_	U_188_–A_171_ G_187_–C_172_	Hassen *et al*.^53^ (double co-variation)
