# Supplementary material for: Interference between variants of peach latent mosaic viroid reveals novel features of its fitness landscape: implications for detection
Source: Sci Rep. 2017 Feb 17;7:42825. doi: 10.1038/srep42825 (PMC5314366; doi:10.1038/srep42825)
Supplement: Supplementary Tables and Figures [file srep42825-s1.pdf]

Interference between variants of peach latent mosaic viroid reveals novel features of its fitness landscape: implications for detection

Pedro Serra[a], Edson Bertolini[b,c], M. Carmen Martínez[b], Mariano Cambra[b] & Ricardo Flores[a]

Table S1. PLMVd primers for RT-PCR, and sets of PLMVd primers and probes for TaqMan rtRT-PCR

Primers[a]	Sequence (5’→ 3’)	Positions[b]	Use
RF43 (c)	CTGGATCACACCCCCCTCGGAACCAACCGCT	207-177	RT-PCR
RF44 (i)	TGTGATCCAGGTACCGCCGTAGAAACT	198-224	RT-PCR
RF1251 (c)	GGACCGGGTTTGAATCCCGGG	260-240	RT-PCR
RF1252 (i)	CCCTCAGAGGTGACTCTGAGTGAAAGG	261-287	RT-PCR
RF1332 (c)	TCCAGTTTCTACGGCGGTACC	227-207	RT-PCR
RF1333 (i)	TTACGACGTCTACCCGGGATTC	228-249	RT-PCR

Primers and probes[a]	Sequence (5´→3´)	Positions[b]	Use
RP1 (c)	ACGGCGGTACCTGGATCA	217-200	rtRT-PCR (reverse primer)
FP1 (i)	TGAGGTAAGGTGGGACTTTTCC	144-165	rtRT-PCR (forward primer)
P1 (i)	CAAGCGGTTGGTTCCGAGGG	175-194	rtRT-PCR (probe)
RP1 (c)	ACGGCGGTACCTGGATCA	217-200	rtRT-PCR (reverse primer)
FP1 (i)	TGAGGTAAGGTGGGACTTTTCC	144-165	rtRT-PCR (forward primer)
P2 (i)	TGAGCGGTCGAACCCAGGGG	175-194	rtRT-PCR (probe)
RP2 (c)	GGGACCGGGWTTGAAT	261-246	rtRT-PCR (reverse primer)
FP2 (i)	CAATGASGTAAGGTGGGACT	141-160	rtRT-PCR (forward primer)
P3 (i)	GGTACCGCCGTAGAAACTGGGTTACG	207-232	rtRT-PCR (probe)

[a](c), complementary; (i), identical
[b]Numbering refers to PLMVd reference variant

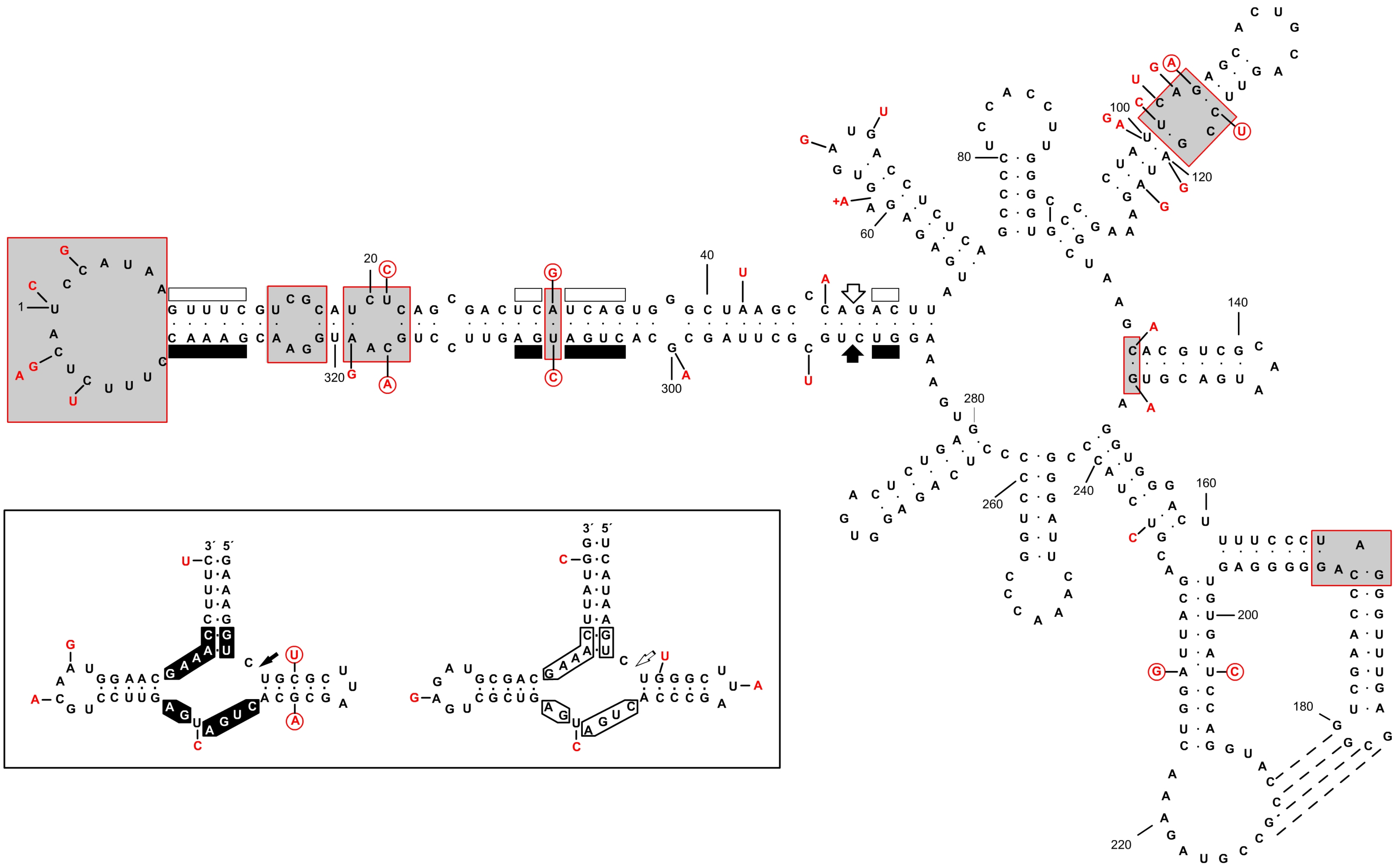

Fig. S1. Sequence variability (in red) detected in two PLMVd isolates of class II (V1 and V2) by RT-PCR using two pairs of adjacent primers of opposite polarity (RF1251 and RF1252). Changes are represented with respect to the primary and secondary structure (generated with Mfold, version 3.4) for the PLMVd plus strand of the reference variant of this class (v1.1) (GenBank KX430152). Local elements of secondary structure that differ from those of the reference variant of classs I are within boxes with a grey background. Nucleotide changes that do not affect the secondary structure are within circles. *Inset*, hammerhead structures of the PLMVd plus and minus strands with the self-cleavage sites marked with arrows. Other details as in Fig. 2, but note that numbering is slightly different because of 1-nt deletion affecting position 167 or 168, and 1-nt insertion after the last position.

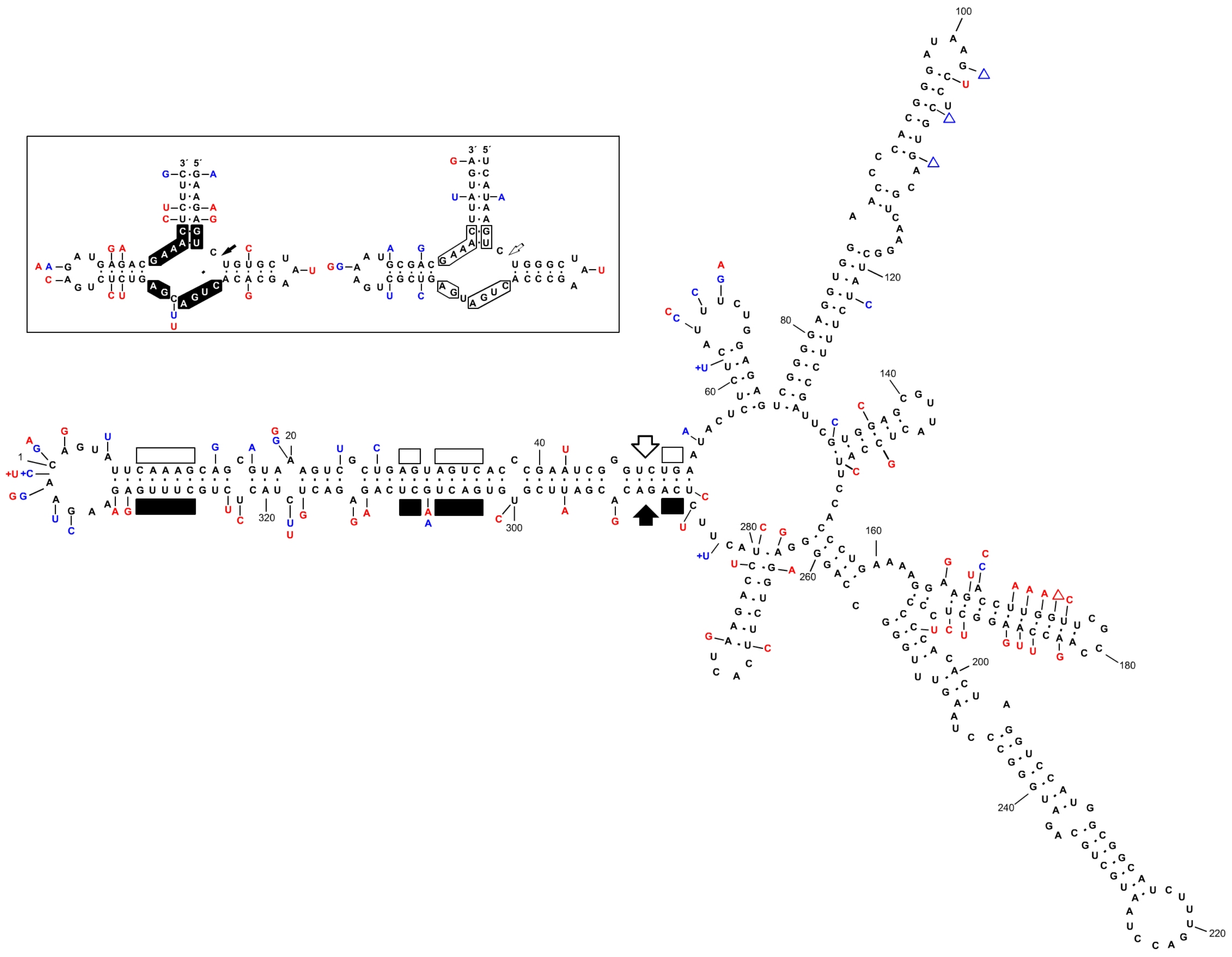

Fig. S2. Primary and proposed secondary structure for the PLMVd minus strand of the reference variant GenBank M83545.1 (Hernández and Flores, 1992) with two minor corrections (Ambrós et al., 1998). Changes in the representative symptomatic variant of class I (gds6) (Ambrós et al., 1998) and in the representative variant of class II (v1.1) (this work) are denoted with blue and red characters, respectively. Numbering is the same as in the plus strand. *Upper inset*, hammerhead structures of the PLMVd plus and minus strands with the self-cleavage sites marked with arrows. Substitutions in variants gds6 and v1.1 do not disrupt the helices flanking the central core of 13 nucleotides (boxed) conserved in most natural hammerhead structures of viroid and viroid-like satellite RNAs. Other details as in Fig 2.
